# Microalgal-bacterial granular sludge: bridging the gap between laboratory discovery and engineering reality

**DOI:** 10.3389/fmicb.2026.1967626

**Published:** 2026-10-28

**Authors:** Bin Ji

**Affiliations:** Department of Water and Wastewater Engineering, School of Urban Construction, Wuhan University of Science and Technology, Wuhan, China

**Keywords:** carbon neutrality, engineering scale-up, microalgal-bacterial granular sludge, resource recovery, wastewater treatment

## Abstract

Microalgal-bacterial granular sludge (MBGS) has been extensively studied as a promising process for energy-efficient and carbon-neutral wastewater treatment. The self-sustaining synergy of this process has been validated under laboratory conditions, and CO₂ supplementation has been shown to push the system toward carbon-negative operation. However, the technology remains largely confined to laboratory benchtops, with most studies conducted under constant conditions using synthetic wastewater. Existing reviews have mainly summarized performance or individual operational factors, but have not translated these findings into a coherent engineering framework for scalable deployment. Based on a comprehensive synthesis of past review articles and experimental studies, this Perspective proposes seven testable and, where possible, quantifiable engineering perspectives: dynamic diel carbon management for net greenhouse gas removal, active microbiome engineering for targeted pollutant degradation, multi-layer stacked photobioreactors with integrated light-guide systems, self-cleaning reactor walls to overcome biofouling, integrated design strategies for simultaneous shear delivery and light utilization in a single reactor, hydrocyclone-based physical selection for long-term stability, and tiered biorefinery with integrated life-cycle assessment. The key advance is not the individual concepts themselves, but their integration into a coordinated, hypothesis-driven framework that shifts the focus from laboratory feasibility to scalable engineering implementation. These perspectives are grounded in established knowledge and offered as testable hypotheses to guide the next generation of research and development toward full-scale deployment.

## Graphical abstract

**Figure:**
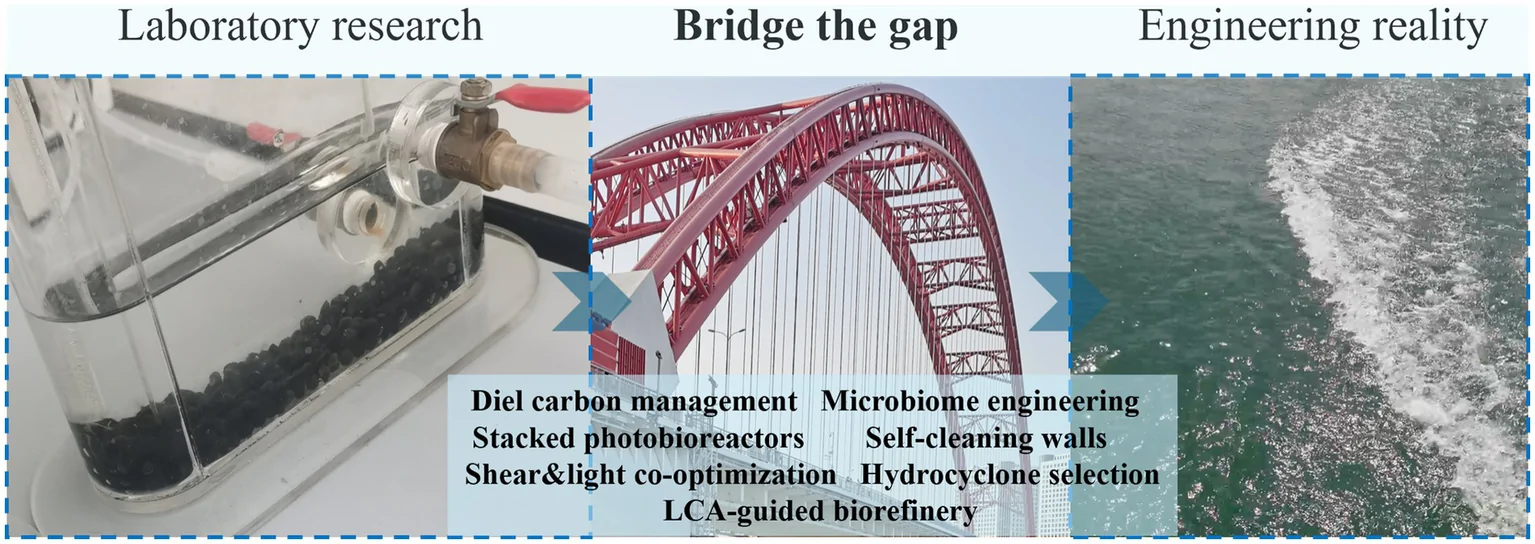

## 1 Introduction

Microalgal-bacterial granular sludge (MBGS) has been increasingly recognized as a promising technology for wastewater treatment. Photosynthetic oxygen production by microalgae can dramatically reduce or even eliminate the need for mechanical aeration (Ji et al., 2020), the single largest energy consumer in conventional activated sludge systems. The dense, fast-settling granular structure enables compact solid‑liquid separation and efficient biomass separation. The algal-bacterial symbiosis, with bacteria typically inhabiting the phycosphere microenvironment surrounding microalgae (Figure 1a), enables simultaneous removal of carbon, nitrogen, phosphorus and a broad spectrum of contaminants within a single granule. In contrast to conventional activated sludge and its upgraded version, aerobic granular sludge, both of which rely on intensive aeration and emit CO₂, MBGS consumes CO₂ through photosynthesis and produces O₂ *in situ* (Figure 1b). Unlike aerobic granular sludge, which still requires mechanical aeration and dissipates pollutants, MBGS couples photosynthesis with granule-based separation, potentially eliminating aeration and concentrating nutrients. The biomass itself is rich in lipids, pigments, and alginate-like exopolymers that can serve as feedstocks for biofuels, bioplastics, and biofertilizers, and other value-added products (Ji and Liu, 2021; Fard and Wu, 2023).

**Figure 1:**
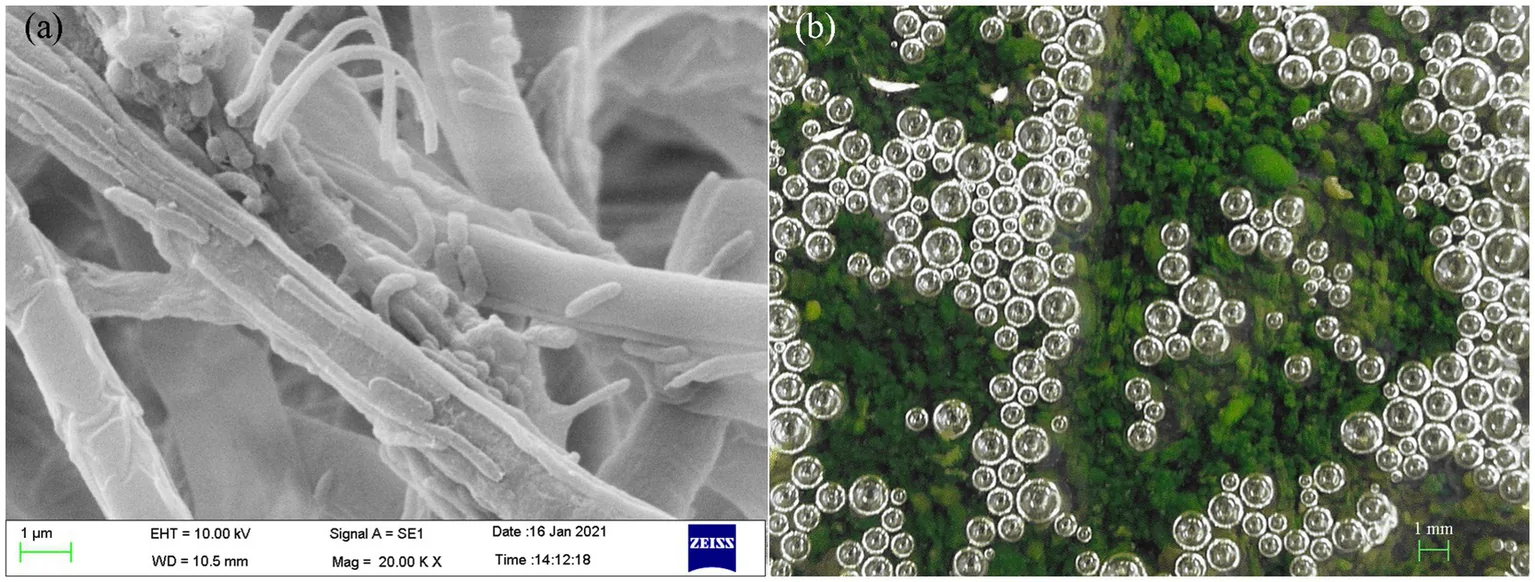
**(a)** SEM image showing rod-shaped bacteria colonizing filamentous microalgae in MBGS; **(b)** oxygen bubbles generated photosynthetically in the non-aerated MBGS reactor.

This technology inverts the core logic of conventional activated sludge. The activated sludge process relies on heterotrophic bacteria, consumes oxygen, emits CO₂, and disperses pollutants. Its upgraded version, aerobic granular sludge, improves settling but still depends on mechanical aeration. MBGS, by contrast, harnesses algal-bacterial mutualism to produce oxygen, fix CO₂, and concentrate dissolved nutrients into dense, fast-settling granules (Ji, 2022). Pollutants are not dissipated but condensed, a shift from net emitter to net absorber, from dissipative to concentrative, from aeration-dependent to light-driven. This inversion indicates that wastewater treatment may fundamentally transform from a net energy consumer into a net energy producer. In conventional activated sludge, aeration typically accounts for the largest share (often 50%) of energy consumption (Liu et al., 2020). MBGS can eliminate this demand under sufficient light and, when powered by photovoltaics, further improve its net energy balance.

The feasibility of a self-sustaining synergistic process without external aeration has been validated (Ji et al., 2020), and CO₂ supplementation has been shown to push the system toward carbon-negative operation (Ji and Liu, 2022). However, MBGS has not achieved meaningful full-scale deployment for mainstream municipal wastewater treatment, and no demonstration projects at the 10 000 m³/d scale have been documented in the open literature (Hammond et al., 2025; Xie et al., 2025). Numerous review studies uniformly draw the consistent conclusion that the technology works well in laboratory settings but faces obstacles during field deployment (Hammond et al., 2025; Khan et al., 2026). The main barriers to large-scale application are threefold: light attenuation in deep reactors severely limits photosynthetic activity; the algal-bacterial consortium is fragile under real-world fluctuations in temperature, light, and influent quality; and the cost of downstream bioproduct purification at commercial scale remains prohibitive.

This Perspective does not rehearse these well-documented challenges. Rather, it offers seven forward-looking perspectives that reframe these obstacles as design opportunities. Each is accompanied by concrete, testable technical pathways. The goal is not to claim that MBGS is ready for immediate deployment, but to articulate what it would actually take to get there.

## 2 Established knowledge and unresolved bottlenecks

Before presenting new perspectives, it is essential to recognize what the past decade of research has firmly established.

MBGS provides a viable path to aeration-free operation. The oxygen self-cycling principle is the foundational advantage of this technology. Microalgal photosynthesis produces sufficient oxygen to sustain aerobic bacterial metabolism. When photosynthetic O₂ production matches or exceeds heterotrophic oxygen demand under sufficient light, mechanical aeration can be fully replaced. Under laboratory conditions, non-aerated MBGS systems have demonstrated excellent COD and nutrient removal (Abouhend et al., 2018; Ji et al., 2020). The mutualistic gas exchange creates a self-sustaining metabolic loop that fundamentally distinguishes this process from conventional activated sludge.

Granulation is a self-organizing ecological process. Mature MBGS granules are highly structured micro-ecosystems. These granules are typically several hundred micrometers to a few millimeters in diameter, settle faster than activated sludge flocs, and allow light penetration mainly in the outer few hundred micrometers. The outer layer, enriched with phototrophic microorganisms, generates oxygen through photosynthesis. The inner layers, depleted of light and oxygen, harbor various microorganisms (Trebuch et al., 2023). This enables simultaneous organics, nitrogen and phosphorus removal within a single granule. Filamentous cyanobacteria act as scaffold engineers, their gliding motility and EPS secretion driving initial aggregation and structural integrity (Milferstedt et al., 2017; Park and Takeuchi, 2021).

The theoretical energy balance favors MBGS over conventional activated sludge. Based on the empirical formula for MBGS biomass, the growth yield is substantially higher than that typical of conventional activated sludge (Ji and Liu, 2021). This translates to greater methane recovery potential via anaerobic digestion. In conventional activated sludge, aeration is the single largest energy consumer, typically accounting for about 50% of in-plant energy consumption (Liu et al., 2020). Eliminating aeration can reduce energy demand by about half, and photovoltaic-powered operation can further improve the net energy balance. Seasonal fluctuations in outdoor light intensity and temperature would substantially reduce the net energy benefit. CO₂ supplementation can enhance this energy balance by increasing biomass productivity and improving methane yields, while simultaneously achieving net carbon removal (Ji and Liu, 2022).

The resource recovery potential is substantial but unproven at scale. MBGS biomass is rich in lipids, extracellular polymeric substances (EPS), and alginate-like exopolymers (ALE), which can be converted to biodiesel, bioplastics, biogas, and biofertilizers (Fard and Wu, 2023; Hao et al., 2025). Representative reported yields include 30–60 mg fatty acid methyl esters (FAMEs)/g SS for lipids, up to 308 mg/g VSS for EPS, and 530 mL CH₄/g VS for methane (Hao et al., 2025). ALE recovery yields range from 13.37 mg/g VSS under standard operation (Chen et al., 2022) to 36.33 mg/g VSS following post‑sampling optimized short‑term cultivation (Chen et al., 2023). However, these values are mostly from laboratory-scale extractions, and methane yields reported in different studies vary considerably. Realistic product yields remain low and variable, limited by biomass composition, extraction efficiency, and downstream processing losses. Industrial-scale recovery faces high energy demand, low selectivity, and micropollutant enrichment risks. The costs of industrial-scale continuous dewatering, cell disruption, and purification equipment have not been systematically assessed.

These four conclusions constitute the solid foundation upon which MBGS research rests. The following seven perspectives are offered as the structural columns required to erect a genuinely deployable technology.

## 3 Seven engineering perspectives toward scalable implementation

### 3.1 Dynamic diel carbon management for net greenhouse gas removal

The literature frames MBGS as low-carbon or carbon-neutral, but existing studies focus separately on daytime photosynthetic carbon fixation or nighttime greenhouse gas emissions (Ji and Liu, 2022). Under proper design, MBGS can be engineered toward net greenhouse gas removal. The diel cycle need not be a liability but can be engineered as a dynamic carbon management system. The carbon balance must therefore account for both daytime and nighttime processes.

During daylight, photosynthesis fixes CO₂ while producing O₂. During darkness, the system may generate greenhouse gases including CO₂, CH₄, and N₂O. However, in closed or semi-closed systems, these gases produced at night can be re-captured upon re-illumination (Sun et al., 2022; Li et al., 2023; Safitri et al., 2021). Specifically, upon re-illumination, photosynthetic CO₂ fixation resumes, while CH₄ and N₂O may be re-assimilated or oxidized by the algal-bacterial consortium, thereby reducing net emissions. Notably, under short HRT conditions, nitrogen removal proceeds primarily through assimilatory uptake, with nitrification–denitrification playing a minor role, reducing N₂O generation (Ji and Liu, 2021). The primary performance metric should be net GHG flux rather than COD and nutrient removal alone. Pilot studies must quantify the diel carbon balance across complete 24-h cycles.

### 3.2 Active microbiome engineering for targeted pollutant degradation

Operational parameters shape consortium structure, but these insights remain largely descriptive. While single-population acclimation cases have been reported, a systematic functional consortium library covering multiple scenarios has not been established. What is needed is prescriptive microbiome engineering: deliberate selection of functional consortia tailored to specific wastewater types.

Indigenous consortia, when properly conditioned, can achieve remarkable performance in challenging wastewaters (Guadalupe et al., 2024; Yu et al., 2024). Extending this to MBGS, a designer consortium approach could pre-adapt communities to specific pollutants before reactor startup. For long-term operation, the desired community can be selected and maintained through controlled selective pressure (e.g., HRT, light/dark cycles), periodic monitoring, and adaptive operational strategies. In practice, three levers are particularly useful: hydraulic selection to retain dense granules, quorum sensing modulation (e.g., boron addition enhancing autoinducer-2 and EPS production), and trace metal dosing (e.g., zero-valent iron) to suppress filamentous overgrowth and enrich functional groups (Ahmad et al., 2017; Wan et al., 2025; Fan et al., 2024). This would shift MBGS from a one-size-fits-all process to a customizable platform. However, community stability under real wastewater remains the main obstacle. The primary causes include light attenuation from turbidity, which disrupts phototroph-heterotroph balance, and seasonal temperature and irradiance fluctuations (Wang et al., 2021). These factors require further investigation.

### 3.3 Multi-layer stacked photobioreactors with integrated light-guide systems

The most persistent criticism of MBGS is that light limitation forces shallow reactors and large land footprints (Chen S. et al., 2025; Hammond et al., 2025). This critique assumes photobioreactors must be single-layer. That assumption can be challenged.

Conventional activated sludge reactors are built below grade, shielding biomass from light while accommodating energy-intensive aeration. MBGS derives its oxygen from photosynthesis, so the reactor must be positioned where light can reach it. This is a logical consequence of the technology’s governing principle.

The technical pathway involves multi-layer stacked photobioreactors with multiple transparent layers, each empirically determined at approximately 0.5 meters deep (Shen et al., 2022), achieving total height comparable to conventional aeration tanks. This depth is chosen based on light attenuation characteristics in dense algal-bacterial aggregates and is consistent with our previous photobioreactor design (Shen et al., 2022). Each layer receives light via passive transmission, light-guide systems distributing sunlight from the roof, and supplementary LED lighting powered by photovoltaics. Mixing within each layer is designed to ensure homogeneous light distribution and prevent dead zones. The reactor concept draws on the raceway configuration, which uses a shallow, elongated channel to maximize light exposure while maintaining continuous circulation (Biliani and Manariotis, 2026). For mixing intensity, a shear stress of approximately 0.18 Pa is required under complex organic conditions to inhibit excessive filamentous growth and maintain granule stability (Wang et al., 2025). The hydraulic retention time is provisionally set at around 6 h (Ji, 2022; Ji and Liu, 2021). For nighttime light supplementation, a 1:1 light-to-dark ratio has been shown to sustain treatment performance during dark phases (Tong et al., 2025). However, reactor dimensions, layer spacing, light-guide layout, and the energy balance of supplemental LED lighting remain unresolved design parameters that require dedicated further research and verification. This can substantially reduce land footprint while maintaining light exposure.

Capital investment is higher than for conventional systems, and economic advantage diminishes where land costs are low. Reactor height imposes structural and maintenance requirements. CO₂ supplementation can enhance algal growth in lower layers. Although supplementary LED lighting adds energy demand, it is powered by photovoltaics, thereby minimizing external energy input. Key validation points include light attenuation measurement, light-guide system performance, and hydraulic distribution in pilot testing. This pathway remains an engineering hypothesis.

### 3.4 Self-cleaning reactor walls to overcome biofouling

Microalgae preferentially attach to reactor walls rather than forming granules (Ueda et al., 2024). Wall biofouling attenuates light penetration and necessitates costly shutdowns of photoreactors (Soriano-Jerez et al., 2024). This should be treated as a design problem.

The technical pathway integrates automated wall-cleaning mechanisms from the outset. Mechanical scraping uses soft wipers on a schedule. Gas or liquid shear uses pulsed high-velocity jets. Antifouling coatings resist adhesion or degrade deposits (Banerjee et al., 2011; Chen Y. et al., 2025). However, quantitative data on cleaning frequency and its associated energy demand remain scarce, and no unified benchmark is currently available. Long-term performance and durability of antifouling coatings remain key uncertainties, including degradation, reapplication, compatibility with cleaning, and possible effects on light transmission, as their efficacy may decrease over time and require periodic reapplication. The robust solution may combine coatings to reduce attachment, supplemented by brief daily shear pulses.

Key validation points include whether cleaning causes granule breakage, durability and cost-effectiveness, and the trade-off between cleaning frequency and light transmittance. This trade-off requires further study. This pathway remains an engineering hypothesis.

### 3.5 Integrated design strategies for simultaneous shear delivery and light utilization

Granule formation requires sufficient shear stress (Liu and Tay, 2002). Effective light supply determines photosynthetic oxygen production. Light penetration depends on reactor configuration and mixing. Light and shear co-optimization is central to reactor design. Under complex organic conditions, 300 rpm is required to inhibit filamentous overgrowth and maintain granule stability (Wang et al., 2025). For scale-up, specific power input or impeller tip speed should serve as the unified control parameter, as the relationship between agitation speed and shear stress is nonlinear (Abouhend et al., 2023).

In most non-aerated configurations, shear and light supply conflict. Aeration bubbles scatter light. Stirrer blades create shadows. Insufficient shear produces loose granules (Wang et al., 2025). Although aeration scatters light, it also enhances mixing and mass transfer; intermittent or micro-aeration can therefore serve as a potential fallback strategy to balance these benefits against light scattering. This trade-off needs to be addressed explicitly.

One direction is integrating light transmission and shear delivery into a single unit. Optical fibers or light-guiding materials in stirrer blades could distribute light while providing shear. This concept builds on existing internally illuminated stirred-tank photobioreactors, where optical fibers are installed within the reactor and function under mechanical agitation (Ogbonna et al., 1999). However, direct experimental evidence for light-emitting stirrers in MBGS systems is currently unavailable, and the concept requires dedicated validation. Challenges include light transmission losses, biofouling, and optical fiber durability. Transparent or reflective stirrer blades with self-cleaning functionality are another avenue.

In the short term, mechanical stirring with optimized speed can provide shear while controlling illumination impact. An optimal stirring window exists where granule integrity remains stable. For scale-up, specific power input or impeller tip speed should be the control parameter. Intermittent or micro-aeration can reduce continuous light scattering. Key validation points include testing stirring speeds and aeration modes on light penetration, particle size, and removal efficiency. This pathway remains an engineering hypothesis.

### 3.6 Hydrocyclone-based physical selection for long-term stability

The most urgent need is pilot-scale validation. Hydrocyclone-based physical selection, proven in aerobic granular sludge, has been overlooked in MBGS. Hydrocyclones separate dense granules from light flocs, returning the former to the reactor and wasting the latter. This is key to stability in full-scale AGS systems like Nereda (Pronk et al., 2015). In AGS systems, hydrocyclones typically achieve high separation efficiency for mature granules, providing a proven basis for adaptation to MBGS.

The pathway integrates a hydrocyclone as a side-stream unit treating a portion of return sludge. Drawing from AGS experience, appropriate cone angle, inlet velocity, and cut size should retain mature granules while discharging light flocs. However, MBGS-specific separation efficiency may be lower due to the surface properties of microalgae, and granule damage may occur during pumping and at the hydrocyclone inlet.

For high-turbidity influents, pre-treatment such as coagulation-flocculation-sedimentation should be integrated to reduce turbidity, ensuring adequate light penetration to the photo-zones. Experience from MBGS operation suggests that pre-treatment should reduce turbidity to levels that allow effective granule photosynthesis and stable system performance. Coagulation-flocculation-sedimentation increases chemical consumption and sludge production, and these additional costs must be weighed against improved light penetration.

Two adaptations are required. First, low-inclination hydrocyclone or reduced feed pressure to mitigate microalgae damage. Studies confirm granule breakage occurs mainly during pumping (Guo et al., 2022), suggesting feed pump optimization can reduce detachment. Second, automated SVI measurement, together with granule size and chlorophyll monitoring for real-time adjustment. A pilot-scale system of adequate capacity should operate long enough to capture seasonal variations. Key validation points include optimizing selection efficiency and assessing microalgae damage. These targets should be prioritized in future pilot studies. This pathway remains a transferable hypothesis requiring MBGS-specific pilot validation.

### 3.7 Tiered biorefinery with integrated life-cycle assessment

Tiered extraction has appeared in multiple reviews, but most studies remain at qualitative description. The core innovation is that life-cycle assessment (LCA) must serve as a design tool throughout process development, not as ex post evaluation. The LCA should clearly define the functional unit (e.g., 1 m^3^ of treated wastewater) and system boundary (from influent to bioproduct recovery and disposal).

The technical pathway involves a four-stage process: high-value extraction of pigments for pharmaceutical applications; EPS and ALE recovery for biopolymer applications (Chen et al., 2023); anaerobic digestion for methane production, enhanced by CO₂ supplementation; and stabilization and fertilization via composting or thermal drying. Representative yields from MBGS and algal-bacterial granular systems include lipid contents up to 205.5 mg/g VSS under air-pulse mixing (Pereira et al., 2025), ALE recovery of approximately 70 mg/g VSS from real wastewater treatment (Zahra et al., 2023), and methane yields of 531 cm^3^/g VS with a methane fraction of 66.2% (Kazimierowicz et al., 2023). These values remain laboratory- or pilot-scale, and industrial-scale yields are likely to be lower due to downstream processing losses.

A basic economic assessment, based on a recent techno-economic study of an integrated algal-bacterial granular sludge and down-flow hanging sponge system for petrochemical wastewater treatment (Ezz et al., 2025), suggests that the combined system can achieve net positive economic returns when biogas and biochar production are included, though this result is not generalizable to standalone MBGS for municipal wastewater. However, this assessment is system-specific, and a full economic evaluation for MBGS remains future work. LCA must guide design decisions. If pigment extraction consumes more energy than the pigments are worth, the process should be redesigned. The LCA boundary must include the entire value chain with sensitivity analysis. Only when positive net environmental and economic balance can be demonstrated is MBGS resource recovery truly sustainable. A basic economic assessment should accompany LCA, and contamination risks (e.g., heavy metals, micropollutants) must be assessed before biomass is used for feed or pharmaceutical applications. Key validation points include standardized extraction protocols. The integration of MBGS with local economic activities depends on context. In urban settings, the reactor itself can be integrated with green building design. In rural or suburban areas, the harvested biomass can be directed to agricultural land as biofertilizer or to aquaculture as feed supplement. This pathway remains an engineering hypothesis.

## 4 A call to action

The seven perspectives shift the discourse from what MBGS can do to what it would take to make it work at scale (Table 1). They are testable hypotheses, not settled truths. Each requires dedicated research effort, some at laboratory scale, others at pilot scale. The path forward is now clear enough to be actionable. A simple roadmap is proposed: (1) laboratory-scale mechanism studies to confirm photosynthetic oxygen supply and identify key functional guilds, with the decision point being stable removal without aeration; (2) pilot-scale component validation to verify light attenuation, mixing, hydrocyclone performance, and antifouling durability, with the decision point being stable operation for more than six months under real conditions; (3) full-scale demonstration with integrated design, monitoring, and LCA to validate energy and carbon balances and biorefinery economics, with the decision point being net energy-positive and carbon-negative operation with economic viability.

**Table 1:** Summary of seven engineering perspectives toward scalable MBGS implementation.

Perspective	Challenge and status	Solution and validation
Diel carbon management	Day/night processes studied separately; darkness may generate CO₂, CH₄, N₂O; short HRT shifts nitrogen removal to assimilatory uptake and reduces N₂O	Engineer diel cycle for net GHG removal; re-capture night gases on re-illumination; use net GHG flux; to be validated: 24-h carbon balance
Microbiome engineering	Insights largely descriptive; no systematic functional consortium library	Prescriptive selection of consortia for specific wastewater; ALE-conditioned indigenous consortia; designer pre-adaptation; customizable platform
Stacked photobioreactors	Light limitation leads to shallow reactors and large land footprint; single-layer assumption; higher capital cost; lower economic advantage where land costs are low; height imposes structural and maintenance needs	Multi-layer approximately 0.5 m transparent layers with light guides and photovoltaic-powered LED; CO₂ supplementation for lower layers; to be validated: light attenuation, light guides, hydraulics
Self-cleaning walls	Wall biofouling attenuates light and causes costly shutdowns	Automated wipers and jets plus antifouling coatings; combine coatings with brief daily shear pulses; to be validated: granule breakage, durability, cost, cleaning–energy–light tradeoff
Shear & light co-optimization	Shear and light conflict: bubbles scatter light, blades shade, low shear produces loose granules	Integrated light-guiding stirrers or blades; transparent or reflective self-cleaning blades; optimized stirring; intermittent or micro-aeration; scale-up by specific power input or impeller tip speed; validate light, particle size, removal; address transmission losses, biofouling, fiber durability
Hydrocyclone selection	Proven in AGS but overlooked in MBGS; urgent pilot validation; high turbidity needs coagulation-flocculation-sedimentation	Side-stream hydrocyclone with optimized cone angle, velocity, cut size; low-inclination or reduced feed pressure; feed pump optimization; online SVI, particle size, chlorophyll monitoring; to be validated: selection and algal damage
LCA-guided biorefinery	Tiered extraction mostly qualitative; LCA should be design tool, not ex post	Four stages: pigments, EPS and ALE, anaerobic CH₄, composting or drying; full-chain LCA with sensitivity analysis; redesign if pigment extraction energy exceeds value; validate standardized extraction; only positive net environmental and economic balance is sustainable

AGS, aerobic granular sludge; ALE, alginate-like exopolymers; EPS, extracellular polymeric substances; GHG, greenhouse gas; HRT, hydraulic retention time; LCA, life-cycle assessment; MBGS, microalgal–bacterial granular sludge; PV, photovoltaic; SVI, sludge volume index.

The remaining challenges are largely engineering problems, which are generally tractable. What is needed is not higher removal rates in the laboratory, but systems that operate stably in the real world. Beyond municipal wastewater, MBGS also holds promise for treating eutrophic surface waters (Trebuch et al., 2024). Natural and engineered granules share a dense, spherical, EPS-rich structure that sustains internal light, oxygen, and nutrient gradients and mutualistic carbon and nutrient exchange. Notably, marimo, a naturally occurring microalgal–bacterial granular consortium found in lake ecosystems, exemplifies such natural granules. The respective roles of natural and engineered granules in lake restoration, urban landscape water maintenance, and wastewater treatment, as well as their potential integration, warrant further investigation.

A division of labor among research actors is proposed. Universities should focus on microbiome engineering mechanisms, anti-fouling materials, and LCA modeling. Equipment manufacturers should lead development and prototyping of core components. Water utilities should take responsibility for year-round outdoor pilot operation and stability validation. A typical municipal wastewater treatment plant could serve as a testbed where hydrocyclone-based stabilization is implemented, universities monitor microbial community evolution, and manufacturers refine reactor configuration based on operational data.

Successful validation of these seven perspectives would enable MBGS to evolve from laboratory curiosity to mainstream technology with potential for energy-positive operation, carbon-negative footprint, and valuable bioproduct recovery. However, these claims require comprehensive validation. A full energy and GHG balance must account for lighting, mixing, pumping, cleaning, hydrocyclone operation, pretreatment, CO₂ supply, and downstream processing. Only after such an integrated assessment can energy-positive and carbon-negative operation be confirmed. The transition requires coordinated effort across disciplines and sectors, but the potential payoff justifies the investment. We invite colleagues to consider these seven perspectives as a starting point for debate, refinement, and experimental validation. The laboratory-to-full-scale gap can be bridgeable. The biological basis is increasingly well established. The remaining challenge is to translate it into scalable, economically viable, and policy-supported systems.

## Data Availability

The raw data supporting the conclusions of this article will be made available by the authors, without undue reservation.
